# The Management and Supervision Tool (MaST): an electronic crisis risk prediction tool to support safe and effective mental healthcare

**DOI:** 10.1192/j.eurpsy.2022.444

**Published:** 2022-09-01

**Authors:** R. Patel, J. Oram, N. Hebden, Z. Payne, M. Morse, C. Gadd

**Affiliations:** 1 King’s College London, Academic Psychiatry, London, United Kingdom; 2 Holmusk, Europe, London, United Kingdom

**Keywords:** Electronic Health Records, Predictive Analytics, CMHT, Crisis

## Abstract

**Introduction:**

The increasing global burden of mental disorders has led to rising demand for mental healthcare services. Effective resource management is essential to ensure safe and timely access to care. Electronic health records (EHRs) provide a real-time source of data on clinical presentation and prognostic factors that could be harnessed to provide clinicians with actionable insights to prioritise mental healthcare delivery. We describe the development and evaluation of MaST, an EHR data visualisation tool that provides information to clinicians on risk of mental health crisis defined as an admission to a psychiatric hospital or acceptance into a community crisis service.

**Figure:**
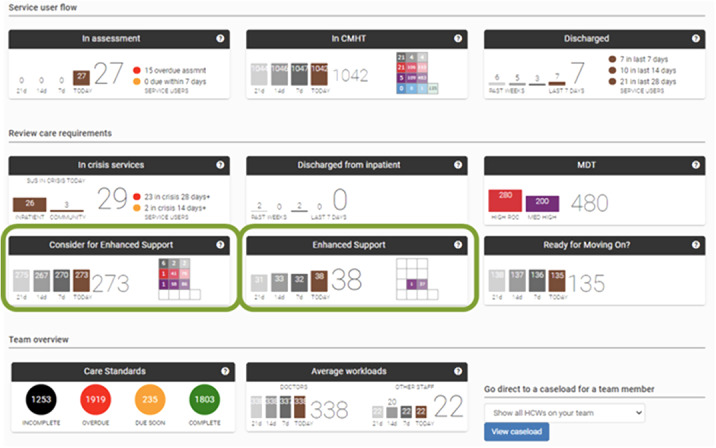

**Objectives:**

(i) To develop an EHR-data driven risk prediction tool for risk of crisis. (ii) To evaluate predictive performance in a real-world clinical setting.

**Methods:**

The risk of crisis algorithm was developed and evaluated with EHR data from six UK NHS mental health providers using Ordered Predictor List propensity scores grouped into 5 quintiles. The predictor variables were clinical and sociodemographic factors including previous mental health service contacts.

**Figure:**
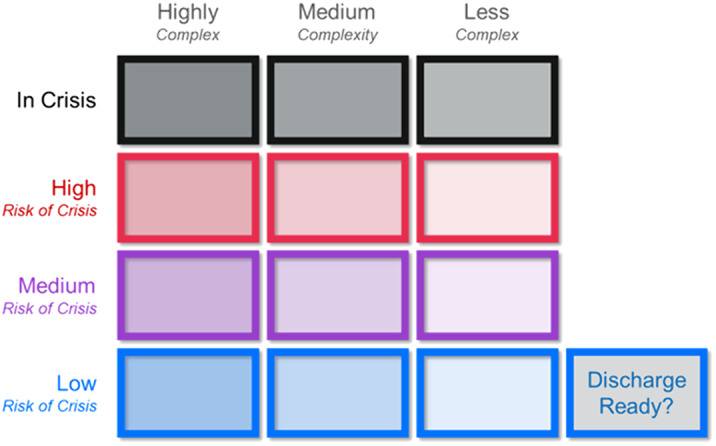

**Figure:**
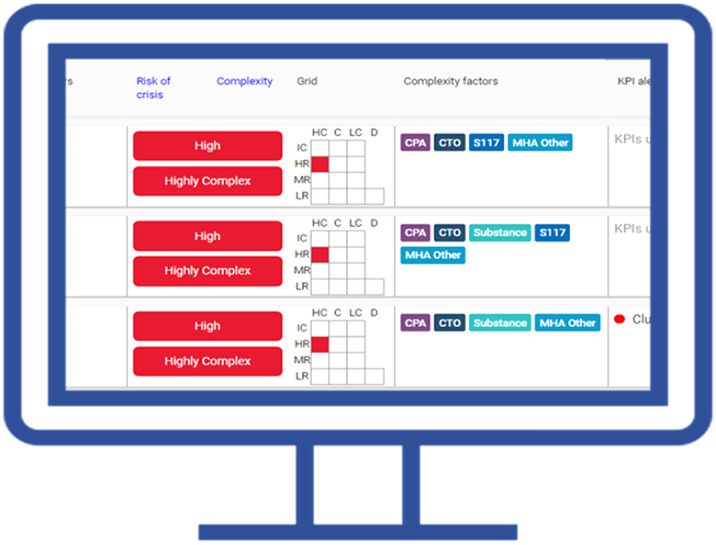

**Results:**

Data from 2,620 patients contributed to algorithm development which was subsequently tested on data from 107,879 patients. The risk of crisis algorithm performed well with an overall accuracy for predicting the greatest risk of crisis (top quintile) ranging from 64% to 80%.

**Conclusions:**

The MaST algorithm accurately predicted risk of mental health crisis in UK community mental health services. EHR data visualisation tools can provide actionable insights to clinicians to prioritise mental healthcare delivery in real-world clinical practice.

**Disclosure:**

This study was funded in full by Holmusk.

